# Awareness of Hepatitis B Among the General Population in Riyadh, Saudi Arabia

**DOI:** 10.7759/cureus.77437

**Published:** 2025-01-14

**Authors:** Talal M Abukaram, Maha Alwan, Abdullah K Alanazi, Sara M Habra, Amanee M Almalik, Sultan S Alanazi, Noura M Alali, Lajeen Alnowaisser, Roaa Alotaibi, Waqar Farooqi

**Affiliations:** 1 Internal Medicine, King Abdulaziz Medical City, Riyadh, SAU; 2 College of Medicine, King Saud Bin Abdulaziz University for Health Sciences, Riyadh, SAU; 3 Family Medicine, Rumah Primary Healthcare Center (PHC), Riyadh, SAU; 4 College of Medicine, International University of Africa, Khartoum, SDN; 5 College of Medicine, Almaarefa University, Diriyah, SAU; 6 College of Medicine, King Faisal University, Al-Ahsa, SAU; 7 Internal Medicine, Almaarefa University, Diriyah, SAU

**Keywords:** awareness, hepatitis b, knowledge, public health, saudi arabia

## Abstract

Background: Hepatitis B, caused by the hepatitis B virus (HBV), remains a significant global public health challenge despite advances in vaccination and treatment. This study aims to evaluate the awareness, knowledge, and perceptions of hepatitis B among the general population in Riyadh, Saudi Arabia.

Methods: A cross-sectional survey was conducted in Riyadh in 2024, involving 805 participants aged 18 years and above. Data were collected using a structured questionnaire that assessed demographic characteristics, general awareness, knowledge of transmission and prevention, testing history, and sources of information. Descriptive statistics and chi-squared tests were used for analysis.

Results: Among the participants, 87% reported awareness of hepatitis B, though detailed knowledge about transmission and prevention revealed gaps. Awareness of blood transfusion as a transmission route was high (83.2%), while unprotected sex (49.3%) and mother-to-child transmission (43.2%) were less recognized. Vaccination was identified as a preventive measure by 83.5%, but misconceptions, such as the efficacy of hand washing (32%), were evident. Only 35.4% of participants had been tested for hepatitis B, highlighting low screening rates. Primary information sources included healthcare providers (57.1%) and the internet (57.5%), with social media cited by 41.5%. Gender, age, and educational background significantly influenced knowledge levels.

Conclusion: While general awareness of hepatitis B is high, critical gaps in detailed knowledge and testing behavior remain. Public health strategies should focus on correcting misconceptions, enhancing screening rates, and leveraging trusted information sources to improve the understanding and management of hepatitis B. This study provides valuable insights for policymakers aiming to bridge these knowledge gaps and enhance disease prevention efforts.

## Introduction

Hepatitis B, caused by the hepatitis B virus (HBV), is a major global health issue that continues to pose significant challenges despite advancements in preventive and therapeutic measures. HBV is a DNA virus that primarily affects the liver, resulting in both acute and chronic infections. Globally, an estimated 296 million people live with chronic hepatitis B, contributing significantly to the burden of liver-related diseases, including cirrhosis and hepatocellular carcinoma [1,2]. The World Health Organization (WHO) has identified hepatitis B elimination as a global priority, emphasizing the need for widespread vaccination, increased public awareness, and improved access to treatment [3,4]. Hepatitis B transmission occurs through exposure to infected blood or bodily fluids, with common routes including perinatal transmission, unsafe injections, unprotected sexual contact, and sharing of needles or other sharp instruments [5,6]. High prevalence rates are often observed in low- and middle-income countries, where limited healthcare resources and insufficient public awareness contribute to the sustained transmission of the virus [7,8]. Among high-risk groups, such as healthcare workers and individuals in close contact with hepatitis B-infected persons, awareness and knowledge about the disease are often inadequate [9,10]. Despite the availability of an effective vaccine since 1982, which has significantly reduced hepatitis B prevalence in many regions, gaps in vaccination coverage remain a significant obstacle to achieving hepatitis B elimination [11,12]. Public health campaigns have been successful in increasing vaccination rates in some countries, but challenges such as vaccine hesitancy, limited healthcare access, and lack of knowledge about hepatitis B persist [13,14]. Antiviral therapies, including nucleoside analogs like tenofovir and entecavir, have been developed to manage chronic hepatitis B infection and prevent its progression to severe liver complications [15,16]. However, many individuals, particularly in resource-limited settings, face barriers to accessing these treatments due to financial constraints, healthcare infrastructure gaps, and social stigma [17,18]. Studies across different populations reveal varying levels of knowledge, attitudes, and practices related to hepatitis B, underscoring the need for tailored interventions to address the specific challenges faced by diverse communities [19,20]. For example, healthcare workers often lack adequate training on HBV prevention and management, while community members may have misconceptions about the disease, leading to stigma and poor health-seeking behavior [21,22]. Addressing these knowledge gaps and promoting widespread screening are critical to identifying infected individuals and linking them to care and treatment [23]. This research aims to provide a comprehensive overview of hepatitis B, focusing on its epidemiology, its transmission dynamics, and the availability of preventive and therapeutic measures. By exploring the current challenges and opportunities in hepatitis B management, this study highlights the urgent need for integrated public health strategies to achieve global hepatitis B elimination goals.

## Materials and methods

Study design and setting

This study is a cross-sectional survey conducted in Riyadh, the capital city of the Kingdom of Saudi Arabia, from September 2024 to January 2025. The target population includes adult men and women aged 18 years and above residing in Riyadh during the study period. The study aims to assess awareness of hepatitis B among the general population.

Sampling method

A quota sampling technique was employed to recruit a representative sample of 805 adults, ensuring diverse representation across age, gender, and educational background. This method was chosen to achieve demographic diversity reflective of the population of Riyadh within the constraints of the study resources and timeframe. While random sampling or stratified sampling could have reduced selection bias, these approaches were not feasible due to logistical challenges, such as the absence of a comprehensive population registry and limited access to certain subpopulations within the study timeframe. Quota sampling allowed for a more pragmatic approach, ensuring the inclusion of key demographic groups essential to address the study objectives. We acknowledge the potential for selection bias and have noted this as a limitation in the "Discussion" section.

Data collection tool

A structured questionnaire was developed after reviewing existing literature [1,2,5,8] and consulting subject matter experts to evaluate participants' awareness, attitudes, and practices regarding hepatitis B. The questionnaire was pretested with a sample of 30 participants from the target population to ensure clarity, comprehensibility, and reliability. Adjustments were made based on feedback to refine the questions for optimal data collection. Reliability testing was conducted using Cronbach's alpha, yielding a value of 0.85, indicating good internal consistency. The questionnaire was also reviewed by three independent experts in public health and epidemiology to validate its content and ensure it aligned with the study objectives. The questionnaire consisted of five sections: (1) Demographic informationcollected data on age, gender, educational background, employment status, and monthly income to understand the population distribution and its impact on awareness and knowledge levels. (2) Awareness assessment evaluated participants' general awareness of hepatitis B, including whether they had heard of the disease and their perceptions regarding the adequacy of community awareness. (3) Knowledge of hepatitis B assessed participants' knowledge of transmission modes, preventive measures (e.g., vaccination, safe practices), and symptoms commonly associated with the disease. (4) Vaccination and testing history examined the history of hepatitis B vaccination and testing among participants, along with their awareness of vaccination availability. (5) Information sources identified the primary sources through which participants obtained information about hepatitis B, such as healthcare providers, the internet, social media, and educational institutions. The questionnaire was designed to be administered in both Arabic and English to accommodate the linguistic diversity of participants.

Data analysis

Participants were categorized as "aware" or "not aware" based on their response to the question: "Have you heard of hepatitis B before?" This question served as the primary indicator for awareness classification, with those answering "yes" classified as "aware" and those answering "no" classified as "not aware." To analyze associations between demographic variables (e.g., age, gender, education level) and awareness levels, chi-squared tests were conducted. This test was appropriate for evaluating relationships between categorical variables. For example, chi-squared tests were used to assess whether awareness differed significantly across age groups, genders, or educational backgrounds. Results were reported with p-values, and a significance level of p < 0.05 was used to determine statistical significance. To further explore patterns in the data, additional descriptive statistics, such as frequencies and proportions, were calculated for all variables, providing a detailed understanding of the distribution of awareness across the study population. While this study focused on binary awareness classification, a detailed analysis of knowledge, attitudes, and practices related to hepatitis B was conducted separately. This analysis considered responses to specific questions on knowledge of transmission modes, prevention methods, symptoms, vaccination history, and information sources. The results of these analyses were presented as percentages and cross-tabulations, providing a comprehensive view of the study findings. Limitations associated with using chi-squared tests alone to assess associations are acknowledged, as they do not control for potential confounding variables. To address this, logistic regression analysis was considered for future studies to identify independent predictors of awareness and reduce the risk of confounding effects.

Ethical considerations

Ethical approval for this study was obtained from the Institutional Review Board (IRB) of Almaarefa University (approval number: IRB24-087) on September 2, 2024. Informed consent was obtained from all participants prior to their inclusion in the study. Confidentiality and anonymity were ensured throughout the research process, and all data were securely stored to prevent unauthorized access.

## Results

The demographic characteristics of the study participants revealed a diverse distribution across various variables (Table 1). The majority of participants were aged 18-24 years (n = 365; 45.3%), followed by those aged 25-34 years (n = 230; 28.6%), with smaller proportions in the age groups of 35-44 years (n = 94; 11.7%), 45-54 years (n = 72; 8.9%), 55-64 years (n = 36; 4.5%), and 65+ years (n = 8; 1%). Women constituted the majority of the sample (n = 491; 61%), followed by men (n = 307; 38.1%), while a small percentage (n = 7; 0.9%) preferred not to disclose their gender. Regarding educational background, most participants held a bachelor's degree (n = 534; 66.3%), followed by those with a secondary school education (n = 189; 23.5%), postgraduate degrees (n = 57; 7.1%), undergraduate degrees (n = 22; 2.7%), and no formal education (n = 3; 0.4%). Monthly income levels showed that 37.6% (n = 303) earned less than 3000SR, 20.1% (n = 162) earned 3000-6000SR, 14.3% (n = 115) earned 6001-9000SR, 10.7% (n = 86) earned 9001-12000SR, and 17.3% (n = 139) earned more than 12000SR. In terms of employment status, 41% (n = 330) were employed, 37.6% (n = 303) were students, 15.3% (n = 124) were unemployed, 5% (n = 40) were retired, and 1% (n = 8) were housewives. These demographic insights provide a detailed overview of the population characteristics represented in the study.

**Table 1:** Demographic characteristics of participants This table presents the demographic distribution of participants, including age, gender, education level, income, and employment status, in frequencies and percentages and provides insights into the diversity of the study sample.

Variable	Options	Frequency	Percentages
Age (in years)	18-24	365	45.3%
	25-34	230	28.6%
	35-44	94	11.7%
	45-54	72	8.9%
	55-64	36	4.5%
	65+	8	1%
Gender	Male	307	38.1%
	Female	491	61%
	Prefer not to say	7	0.9%
Educational background	Secondary school	189	23.5%
	Bachelor's degree	534	66.3%
	Postgraduate degree	57	7.1%
	Undergraduate degree	22	2.7%
	No formal education	3	0.4%
Monthly income	Less than 3000SR	303	37.6%
	3000-6000SR	162	20.1%
	6001-9000SR	115	14.3%
	9001-12000SR	86	10.7%
	More than 12000SR	139	17.3%
Employment status	Student	303	37.6%
	Employed	330	41%
	Unemployed	124	15.3%
	Retired	40	5%
	Housewife	8	1%

The findings related to awareness, knowledge, perception, and information sources for hepatitis B among participants are summarized as follows: The majority of participants (n = 700; 87%) had heard of hepatitis B, while 13% (n = 105) had not. When asked if awareness of hepatitis B was sufficient in their community, 35.3% (n = 284) agreed, 48.3% (n = 389) disagreed, and 16.4% (n = 132) were unsure. Regarding knowledge of hepatitis B transmission, 83.2% (n = 670) were aware of blood transfusion as a mode of transmission, followed by sharing needles (n = 489; 60.7%), unprotected sex (n = 397; 49.3%), and mother-to-child transmission (n = 348; 43.2%). Only 12.5% (n = 101) believed it could be transmitted through casual contact, while 5% (n = 40) did not know any modes of transmission. Preventive measures were recognized by many, with 83.5% (n = 672) acknowledging vaccination, 62.9% (n = 506) mentioning avoiding needle sharing, and 40.2% (n = 324) citing condom use. However, 32% (n = 258) believed regular hand washing could prevent hepatitis B, while 2.1% (n = 17) did not know any preventive measures. In terms of symptoms, jaundice was the most recognized (n = 570; 70.8%), followed by fatigue (n = 473; 58.8%), dark urine (n = 383; 47.6%), nausea and vomiting (n = 357; 44.3%), and abdominal pain (n = 350; 43.5%). Persistent cough was less commonly associated with hepatitis B (n = 134; 16.6%), and 3.1% (n = 25) reported no knowledge of symptoms. For vaccination and testing history, 35.4% (n = 285) reported having been tested for hepatitis B, while 64.6% (n = 520) had not. Awareness of vaccination availability in Saudi Arabia was reported by 57.4% (n = 462), whereas 42.6% (n = 343) were unaware. The primary sources of information about hepatitis B were the internet (n = 463; 57.5%) and healthcare providers (n = 460; 57.1%). Social media was cited by 41.5% (n = 334), educational institutions were cited by 36.4% (n = 293), family and friends were cited by 24.7% (n = 199), and television was cited by 16.9% (n = 136). These results highlight gaps in awareness and knowledge, as well as the importance of diverse information sources for disseminating knowledge about hepatitis B (Table 2).

**Table 2:** Awareness, knowledge, perceptions, and information sources related to hepatitis B among participants This table displays participants' awareness, knowledge, and perceptions of hepatitis B, along with information sources, in frequencies and percentages. KSA: Kingdom of Saudi Arabia

Variables	Questions	Options	Frequency	Percentages
Awareness of hepatitis B	Have you heard of hepatitis B before?	Yes	700	87%
		No	105	13%
	Do you think hepatitis B awareness is sufficient in your community?	Yes	284	35.3%
		No	389	48.3%
		Not sure	132	16.4%
Knowledge of hepatitis B transmission and prevention	Which of the following modes of transmission are you aware of?	Blood transfusion	670	83.2%
		Unprotected sex	397	49.3%
		Sharing needles	489	60.7%
		Mother-to-child	348	43.2%
		Causal contact	101	12.5%
		I don't know	40	5%
	How can hepatitis B be prevented?	Vaccination	672	83.5%
		Condoms	324	40.2%
		Avoid sharing needles	506	62.9%
		Regular hand washing	258	32%
		Avoid contact with infected people	322	40%
		I don't know	17	2.1%
Knowledge of hepatitis B symptoms	Which of the following symptoms are associated with hepatitis B?	Jaundice	570	70.8%
		Fatigue	473	58.8%
		Abdominal pain	350	43.5%
		Dark urine	383	47.6%
		Nausea and vomiting	357	44.3%
		Persistent cough	134	16.6%
		I don't know	25	3.1%
Vaccination history	Have you ever been tested for hepatitis B?	Yes	285	35.4%
		No	520	64.6%
	Are you aware of the availability of hepatitis B vaccination in KSA?	Yes	462	57.4%
		No	343	42.6%
Source of information	Where do you usually obtain information about hepatitis B?	Healthcare providers	460	57.1%
		Internet	463	57.5%
		Television	136	16.9%
		Social media	334	41.5%
		Family and friends	199	24.7%
		Educational institutions	293	36.4%

The results presented in Table 3 include chi-squared values and p-values for each demographic variable, with chi-squared tests (χ²) employed to assess associations. A p-value of <0.05 was considered statistically significant. Among the variables analyzed, gender demonstrated a statistically significant association with awareness (p < 0.001), with women exhibiting higher awareness levels (61.9%) compared to men (37.3%). In contrast, age did not show a statistically significant association with awareness (p = 0.306). Although participants aged 18-24 years accounted for the largest proportion of aware individuals (45.6%), followed by those aged 25-34 years (28.7%), the differences were not statistically significant. Similarly, education level was not significantly associated with awareness (p = 0.107), with the majority of aware participants holding a bachelor's degree (66.6%), followed by secondary school graduates (23.6%) and those with postgraduate degrees (7.1%). Monthly income also did not demonstrate statistical significance (p = 0.076), despite participants earning less than 3000SR forming the largest aware group (37.1%), followed by those earning 3000-6000SR (19.9%) and 6001-9000SR (14.1%). Employment status showed no significant relationship with awareness (p = 0.889), with employed participants (41%) and students (37.9%) having the highest awareness rates, while housewives had the lowest (0.9%). Overall, gender was the only demographic variable significantly associated with awareness, whereas age, education, income, and employment status did not show statistically significant associations.

**Table 3:** The relation between demographic data and knowledge of participants This table demonstrates the associations between demographic variables (age, gender, education, income, and employment) and awareness of hepatitis B in frequencies and percentages. The p-value of gender is the only significant data.

Variable	Options	Aware "n (%)"	Not aware "n (%)"	Chi-squared test	P-values
Age (in years)	18-24	319 (45.6)	46 (43.8)	0.99	P = 0.306
	25-34	201 (28.7)	29 (27.6)		
	35-44	82 (11.7)	12 (11.4)		
	45-54	63 (9)	9 (8.6)		
	55-64	31 (4.4)	5 (4.8)		
	65+	4 (0.6)	4 (3.8)		
Gender	Male	261 (37.3)	46 (43.8)	10.25	P = 0.001
	Female	433 (61.9)	58 (55.2)		
	Prefer not to say	6 (0.9)	1 (0.9)		
Educational background	Secondary school	165 (23.6)	24 (22.9)	6.13	P = 0.107
	Bachelor's degree	466 (66.6)	68 (64.8)		
	Postgraduate degree	50 (7.1)	7 (6.7)		
	Undergraduate degree	19 (2.7)	3 (2.9)		
	No formal education	2 (0.3)	1 (1)		
Monthly income	Less than 3000SR	260 (37.1)	43 (41)	6.84	P = 0.076
	3000-6000SR	139 (19.9)	23 (21.9)		
	6001-9000SR	99 (14.1)	16 (15.2)		
	9001-12000SR	74 (10.6)	12 (11.4)		
	More than 12000SR	128 (18.3)	11 (10.5)		
Employment status	Student	265 (37.9)	38 (36.2)	0.21	P = 0.889
	Employed	287 (41)	43 (41)		
	Unemployed	107 (15.3)	17 (16.2)		
	Retired	35 (5)	5 (4.8)		
	Housewife	6 (0.9)	2 (1.9)		

## Discussion

This study provides critical insights into the awareness, knowledge, and perceptions of hepatitis B among the general population in Riyadh, Saudi Arabia. The findings, compared to international studies, highlight significant and non-significant results, emphasizing areas for improvement. The study found that 87% of participants were aware of hepatitis B, aligning with studies conducted in France (85%) [1] and Malaysia (84.5%) [8].

However, despite this high general awareness, detailed knowledge about transmission and prevention was lacking, echoing findings from Pakistan [4] and Jordan [11]. Blood transfusion was identified as a transmission mode by 83.2% of participants, comparable to data from China (84%) [5], but awareness of other modes, such as unprotected sex (49.3%) and mother-to-child transmission (43.2%), was lower than findings from Hong Kong (63%) [7] and Cameroon (56%) [18], indicating a need for targeted education addressing less recognized transmission routes.

Vaccination was correctly identified as a preventive measure by 83.5%, consistent with Southeast Asian studies (80%) [13], but 32% believed regular hand washing could prevent hepatitis B, reflecting misconceptions similar to findings from Pakistan [4] and Jordan [11], highlighting the necessity for public health campaigns to correct misinformation. Only 35.4% of participants had been tested for hepatitis B, consistent with low screening rates reported in Pakistan [6] and Cameroon [18], potentially due to financial barriers, lack of access, or social stigma [17], suggesting integrating free or subsidized testing programs to improve early detection.

Healthcare providers (57.1%) and the internet (57.5%) were the primary information sources, but 41.5% cited social media, posing misinformation risks, mirroring findings in British Columbia [23], and necessitating educational campaigns focusing on digital literacy. Symptoms such as jaundice (70.8%) and fatigue (58.8%) were commonly identified, aligning with Southeast Asian findings [13], but fewer participants recognized dark urine (47.6%) and nausea and vomiting (44.3%), showing gaps similar to results from Ghana [20], emphasizing the need for symptom-focused education to promote early diagnosis.

Gender showed a significant association with awareness (p < 0.001), with women exhibiting higher awareness, contrasting studies in Turkey with minimal gender differences [22], while age, education, income, and employment status did not show significant associations (p > 0.05), consistent with findings from Malaysia [8] and Poland [17], suggesting that awareness campaigns should target all demographics equally.

Recommendations include addressing misconceptions through public education [4,11], enhancing screening rates by integrating testing into routine health services [18], leveraging healthcare providers as trusted sources [2,22], improving digital literacy to regulate health content on social media [23], and implementing community awareness programs modeled after the San Francisco Hep B Free Campaign [17], to address stigma and promote testing.

This study confirms previous findings while highlighting region-specific gaps in awareness and knowledge, reinforcing the importance of targeted interventions to improve screening rates, correct misconceptions, and enhance information dissemination through credible sources, enabling policymakers and healthcare providers to design effective public health strategies aimed at hepatitis B prevention and control.

Limitations

While this study provides valuable insights into awareness and knowledge of hepatitis B, certain limitations should be acknowledged. The findings are based on data collected in Riyadh, which may limit their generalizability to other regions or populations. Additionally, the reliance on self-reported questionnaires may have introduced recall bias or social desirability bias, potentially affecting response accuracy. The study focused on assessing awareness and knowledge without evaluating actual preventive behaviors, which may require further exploration. Moreover, some demographic groups, such as older adults and individuals with lower education levels, were underrepresented, limiting the broader applicability of the results. Future research should address these limitations by adopting longitudinal designs, expanding the geographic scope, and integrating qualitative methods to gain deeper insights into attitudes and behaviors related to hepatitis B.

## Conclusions

This study highlights both the successes and challenges in the awareness, knowledge, and perceptions of hepatitis B among the general population in Riyadh. While general awareness of hepatitis B was found to be relatively high, detailed knowledge regarding its transmission, prevention, and symptoms revealed notable gaps. These findings underscore the need for targeted public health interventions to address misconceptions, improve knowledge accuracy, and promote routine testing and vaccination. The low testing rates and misconceptions about prevention methods indicate barriers that need to be addressed through improved healthcare access, community-based initiatives, and tailored educational programs. Moreover, the reliance on diverse information sources, such as healthcare providers, the internet, and social media, calls for a strategic approach to ensure the accurate dissemination of health information and counter misinformation. By leveraging the strengths of existing public health campaigns and addressing the identified gaps, policymakers and healthcare stakeholders can enhance the effectiveness of hepatitis B management programs in Saudi Arabia, ultimately contributing to the global effort to eliminate this disease.
